# Prolonged Refractory Hypotension following Combined Amlodipine and Losartan Ingestion Responsive to Metaraminol

**DOI:** 10.1155/2011/283672

**Published:** 2011-05-12

**Authors:** James O. M. Plumb, Craig Stewart, Michael Eddleston, Thearina de Beer

**Affiliations:** ^1^Department of Intensive Care Medicine, Nottingham University Hospitals Trust, Queen's Medical Centre Campus, Derby Road, Nottingham NG7 2UH, UK; ^2^Department of Elderly Medicine Royal Derby Hospital, Derby DE22 3NE, UK; ^3^Clinical Pharmacology Unit, University of Edinburgh, Edinburgh EH4 2EH, UK; ^4^National Poisons Information Service-Edinburgh, Royal Infirmary of Edinburgh, Edinburgh EH16 4SA, UK

## Abstract

*Introduction*. Overdose with the calcium channel blocker amlodipine can cause profound hypotension that may be exacerbated by the concurrent ingestion of an angiotensin II receptor antagonist. Best management of such overdoses is uncertain although the use of hyperinsulinaemia-euglycaemia (HIE) has been recommended. *Case report*. We report a case of mixed amlodipine and losartan overdose in a 50-year-old lady. Severe hypotension was resistant to conventional vasopressors and high-dose insulin/euglycaemia, but did respond to a metaraminol infusion. *Conclusion*. A trial of metaraminol early in severe cases of calcium channel blocker and angiotensin II receptor antagonist toxicity may be of benefit, especially when conventional ionotropic treatment measures are failing.

## 1. Introduction

Severe calcium-channel blocker overdose of the dihydropyridine class has a profound effect on the systemic vascular resistance. Of the dihydropyridine class, amlodipine is often favoured for its once daily dosing, lack of negative ionotropy, and prolonged duration of effect (which can last up to 72 hours) [1]. Conventional treatment of calcium channel blocker overdose centres on increasing transmembrane calcium flow either by increasing extracellular calcium concentration or by increasing intracellular cAMP concentration, which can be achieved by adenylate cyclase stimulation (adrenaline or glucagon) or phosphodiesterase inhibition (amrinone, milrinone) [2]. However, no controlled trails have been conducted, and treatment successes or failures have been reported in almost equal measure [3–8]. 

Angiotensin II receptor blockers (in this case Losartan) bind at the AT1 receptor and inhibit vasoconstriction, sympathetic activation, peripheral noradrenergic transmission, baroreceptor desensitisation, endothelin release, renal sodium reabsorption, adrenal cortical aldosterone release, and nitrous oxide destruction [9, 10].

The combination of an angiotensin II receptor blocker and a calcium channel blocker could produce a synergistic toxicity by limiting the effectiveness of both endogenous and exogenously administered catecholamines [11].

Treatment for a mixed overdose in these patients have included: intravenous fluid guided by central venous pressure, calcium chloride/gluconate boluses/infusion, glucagon boluses or infusion, atropine, isoprenaline, high dose noradrenaline/adrenaline, vasopressin/terlipressin, haemoperfusion, hyperinsulinaemia-euglycaemia, and intralipid. [3–8, 11–17].

Hyperinsulinaemia-euglycaemia in the treatment of severe calcium channel blocker overdose is not without controversy. Its effectiveness has never been rigorously established [14]. It has been proposed that failure of hyperinsulinaemia-euglycaemia in severe calcium channel blocker overdose has sometimes been due to inadequate dosing and speed of starting treatment [2, 14].

To our knowledge, only one previous case of severe calcium channel blocker and angiotensin II receptor blocker overdose has been reported in the literature; this reported success with hyperinsulinaemia-euglycaemia, although the data in the paper's figures is not convincing [11]. We report a case of severe mixed amlodipine and losartan overdose causing refractory hypotension resistant to all conventional vasopressors and hyperinsulinaemia-euglycaemia, but responsive to a metaraminol infusion.

## 2. Case Report

A 50-year-old lady presented to the emergency department (ED) having called the ambulance herself. She reported taking an overdose (handfuls of tablets) of her blood pressure tablets 2 h 45 min previously. The empty packets that she brought with her equated to 104 × 160 mg of losartan (16,640 mg) and 77 × 10 mg of amlodipine (770 mg). She had a past medical history of type 2 diabetes mellitus, hypertension, and morbid obesity (weight 137 kg). 

At presentation, her airway was patent, with a respiratory rate of 14 breaths/minute and SpO_2_ of 93% in room air, pulse 100 beats/minute, blood pressure 122/61 mmHg, and temperature 36.4°C, with Glasgow Coma Score of 15/15. Her ECG was normal. 

Her initial laboratory results revealed a normal full blood count (FBC), normal renal function including normal potassium, normal liver function tests, and a blood glucose of 10.6 mmol/litre.

**Figure 1:**
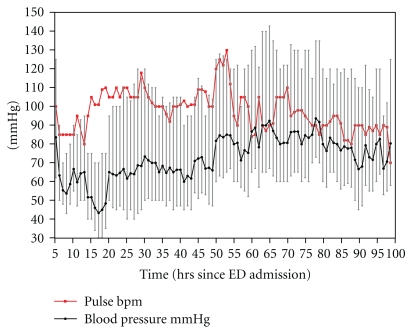
Haemodynamic parameters over the intensive care period.

**Figure 2:**
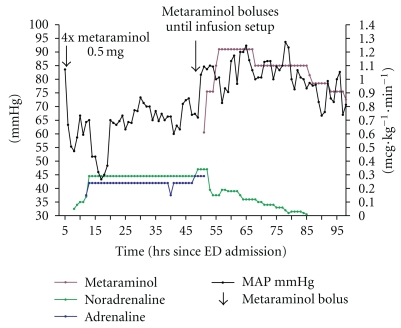
Response of MAP in mmHg to infusions over the intensive care period. Note: left *y*-axis MAP in mmHg, right *y*-axis infusions of ionotropes in mcg·kg^−1^·min^−1^. Arrows show metaraminol boluses.

Shortly after initial assessment in the ED, her blood pressure dropped rapidly to 70/40 mmHg with a pulse of 90 beats/minute that was unresponsive to resuscitation with 2 litres of IV fluid. A central venous line was inserted, and she was transferred to Medical High Dependency. A further 3 litres of intravenous fluid were administered, but she remained anuric and her blood pressure remained at 75 mmHg systolic (mean arterial pressure (MAP) 45–50 mmHg (see Table 1 and Figures 1 and 2). An arterial line was inserted.

Five hours after presentation in the ED, an infusion of noradrenaline (0.1 mcg·kg^−1^·min^−1^) the maximum allowed on High Dependency, was started. As this was being setup, metaraminol (boluses of 0.5 mg) were administered producing a transient increase in MAP (Table 1, Figure 2). She also received 20 mLs of 10% calcium gluconate. At this time, pulmonary oedema was seen on X-ray and hypoxia noted (SpO_2_ 91% on FiO_2_ of 60%). She was started on noninvasive ventilation (NIV) with improvement in oxygenation. Her cognitive function remained normal throughout with a GCS of 15.

Advice from the UK National Poisons Information Service at this time was to: give further calcium and start an infusion, try glucagon, continue to uptitrate the ionotropes and consider terlipressin or vasopressin, and to start hyperinsulinaemia-euglycaemia therapy (1 unit/kg bolus then infuse at 0.5 units/kg/hr, increasing to a maximum of 10 units·kg^−1^·hr^−1^). 

Glucagon was given as a bolus of 10 mg followed by an infusion once the patient arrived on ICU. The first bolus did appear to improve the MAP to above 80 mmHg in much the same way as the metaraminol had; however, this was also transient and caused severe vomiting. 

She was transferred to intensive care 11 hours after presentation to the ED where she was started on vasopressin and the noradrenaline was uptitrated initially to 0.05 mcg·kg^−1^·min^−1^ and then 0.29 mcg·kg^−1^·min^−1^ within the first hour with continued increases over the day. Due to a lack of response, adrenaline was also added along with a calcium infusion (Figure 2). 

Hyperinsulinaemia-euglycaemia was started at 14 hours after admission; after a bolus, the infusion was increased to 6 U·kg^−1^·hr^−1^ over the next 12 hrs without any sustained improvement in blood pressure, although there was a slight rise in MAP over the initial period. At 14 hrs after admission to the ED, she was administered 150 mL bolus of intralipid 20% over 5 min and an infusion of 3 mL·kg^−1^·hr^−1^ was then started (650 mL given in total). Twenty hours after admission to the ED, sodium bicarbonate (200 mLs of 8.4%) was administered; again without response haemodynamically but with an improvement in the acid base status. By this point, she had a severe metabolic acidosis (see Table 2).

At this time (18 hrs after presentation to ED, Figures 1, 2, 4, and 5), she was refractory to vasopressin (0.02 units·hr^−1^), adrenaline (0.24 mcg·kg^−1^·min^−1^), noradrenaline (0.29 mcg·kg^−1^·min^−1^), glucagon (0.12 units hr^−1^), hyperinsulinaemia-euglycaemia (800 units hr^−1^), and calcium (3 mL·kg^−1^·hr^−1^ of 10%). She remained anuric and her renal function was deteriorating (peak creatinine 231 mmol·l^−1^ and urea 14.6 mmol·l^−1^) (at 48 hrs after admission to ED)

The patient was started on a metaraminol infusion 46-47 hrs after admission to ED following a discussion with the team about her initial response to the drug. There was an immediate striking response, the MAP increasing from below 70 mmHg to more than 80 mmHg for the first time since admission (aside from the boluses given at 5 hours), but even more striking was the change in urine output which increased dramatically (Figure 5). The effect of metaraminol permitted the other ionotropes to be weaned over the next few hours (Figure 2). Her urine output improved but she developed hyperkalaemia (6.3 mmol·l^−1^)—a likely side effect of hyperinsulinaemia-euglycaemia therapy cessation.

She made a slow recovery and was discharged to Medical High Dependency 6 days after admission. She was discharged from hospital, after psychiatric input, shortly afterwards.

## 3. Discussion

This is the first case report of severe calcium channel blocker and angiotensin II receptor blocker overdose that was successfully treated with a metaraminol infusion where other treatment options failed or had not been tried. The only other case report in the literature did not use metaraminol by infusion, as the drug was not available to them. Direct personal communication with the lead author confirmed this [11]. The paper by Wood et al. which successfully used metaraminol did not use hyperinsulinaemia-euglycaemia at all and the overdose, although mixed, had a much lower (120 mg) dose of an angiotensin converting enzyme inhibitor compared with the 16.6 g of the angiotensin II receptor blocker that the patient in our case had ingested [4]. 

Hyperinsulinaemia-euglycaemia appears to work by allowing the switch of cellular metabolism from fatty acids to carbohydrates that is required in stress conditions, especially in the myocardium and vascular smooth muscle, resulting in an improvement in cardiac contractility and restored peripheral resistances [2].

Most of the current evidence around hyperinsulinaemia-euglycaemia (from the various case reports quoted) seems to suggest that starting it early is crucial to its success. We started it within 14 hours. There is not a consistent time frame in the literature that constitutes early administration. However, as described in a couple of other case reports, we found that it had little effect [12, 15]; however, there was perhaps a transient increase at the start when we were infusing 800 units per hour; however, multiple infusions were initiated between 11 and 16 hours so it is difficult to attribute this to the hyperinsulinaemia-euglycaemia and the ionotropes were greatly uptitrated during this period (Figure 4).

In our case, we did however find that keeping up with the blood sugar was problematic and dangerous. Large amounts of glucose were required with intensive nursing care to prevent hypoglycaemia. We found that the glucose suddenly dropped over the space of 3 minutes between checks after a long period of not changing consistent with the reported 40 minutes or so for the hyperinsulinaemia-euglycaemia to start working. Previous case reports and reviews have mentioned the low side effects of hyperinsulinaemia-euglycaemia making it an attractive option [2].

It was unclear why glucagon appeared to have a transient effect in our case. It did, however, cause severe vomiting. Vomiting is a well-documented side effect of glucagon, particularly in high doses as given in this case. 

Intralipid was also tried. Due to amlodipine being highly lipophilic, it would seem plausible that intralipid would work to bind it. Young et al. reported a case of verapamil overdose that resulted in cardiac arrest that was successfully treated with intralipid [18]. We could not find any other case reports of significant calcium channel blocker/angiotensin II receptor blocker overdose where intralipid was used but TOXBASE advises that “If life threatening arrhythmias (e.g., ventricular tachycardia, fibrillation, heart block, and asystole) are unresponsive to the above consider the use of a lipid emulsion (e.g., 1 mL/kg of 20% intralipid as an intravenous bolus followed by 3 mL/kg/hour to an initial maximum of 500 mL lipid in an adult).” This did not appear to have any clinical effect. It is unclear if it would have been more effective if it had been given earlier in the clinical course. There is weak evidence for the use of intralipid in verapamil overdose [16]. However, overall the evidence for the use of intralipid in overdose is limited to case reports and animal studies and although promising results have been reported the current guidance for its use is limited to individual agents. For example, the Association of Anaesthetists of Great Britain and Northern Ireland (AAGBI) recommend its use for local anaesthetic toxicity, perhaps the group of drugs where the greatest body of anecdotal evidence exists.

## 4. Why Did Metaraminol Work When All Else Failed?

It is unclear why a synthetic, predominantly alpha-agonist (metaraminol) should have such a profound effect when other agents with alpha-1 effects, most notably noradrenaline did not seem to. Whether it is the mechanism of action, a direct alpha agonist and an indirect alpha and beta agonist (via noradrenaline and adrenaline), or whether it has a property that has not yet been elucidated is speculative. The end product of metaraminol and other predominantly alpha-1 agents is calcium, but this is due to inositol triphosphate (which can act extracellularly), due to an increase in phospholipase C rather than from increased cAMP that results from adrenaline or noradrenaline. This additional pathway may go some way to explain why metaraminol worked.

It has been recognised that pure alpha agonists such as phenylephrine and methoxamine have a role in the management of anaphylaxis where adrenaline has failed to have an effect. Most notably in a case of anaphylaxis during open cardiac surgery where the ionotropic and chronotropic effects were visible to the authors but the lack of SVR and underfilling meant that phenylephrine was crucial to the successful management of the patient [19].

Another reason why metaraminol may have worked where other agents did not is the possibility of dynamic left ventricular outflow obstruction. The diagnosis in critical care is important as reducing catecholamines and adding vasopressing agents may actually improve the situation [20]. 

**Figure 3:**
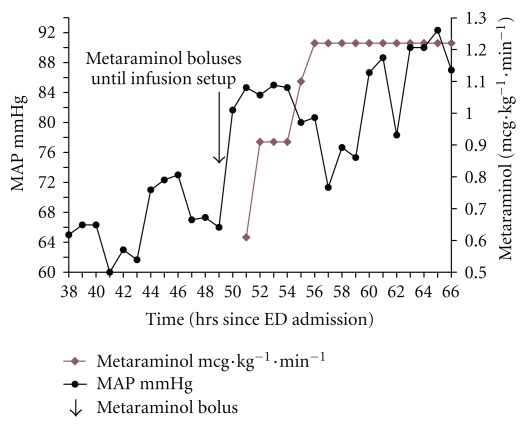
MAP after starting metaraminol infusion (hours 38–66). Note left *y*-axis MAP in mmHg, right *y*-axis metaraminol in mcg·kg^−1^·min^−1^. Arrows show metaraminol boluses.

**Figure 4:**
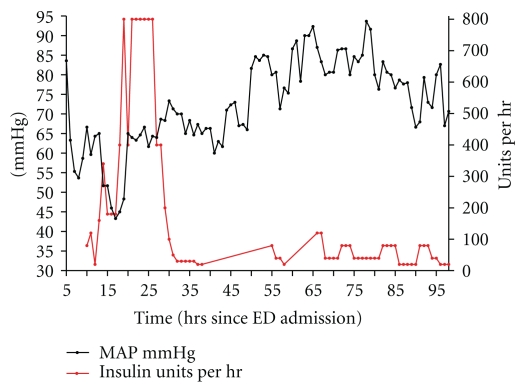
The effect of hyperinsulinaemia-euglycaemia on MAP.

**Figure 5:**
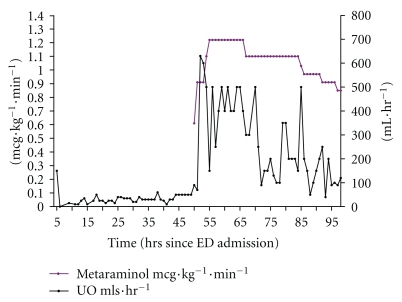
The effect of metaraminol on urine output.

The metaraminol infusion was started later in the course of this case, and it could be proposed that the effect of the calcium channel blocker and angiotensin II receptor blockers were wearing off by this time. Once-daily administration of losartan is possible because the drug's effects are extended by the EXP-3174 metabolite (which has a half-life of 6–9 hours), which is 40 times more potent than losartan and has been found to produce consistent reductions in blood pressure over a 24-hour period. [21] However, the half-life of amlodipine is 35–50 hours although the effects can persist for significantly longer [1]. A striking change was seen when metaraminol was given at the very start of the case (Table 1) and then again when it was bloused and started as an infusion (Figures 2 and 3). The patient was weaned from all other ionotropes within 2 hours (Figure 2). 

Conventional methods have often failed to improve haemodynamics in severe overdoses of this kind and *Lheureux* mentions in his excellent review that people have resorted to extreme measures such as intra-aortic balloon counterpulsation and extracorporeal circulatory support [2]. We propose a less extreme measure (the use of metaraminol by infusion) that is readily available and relatively cheap and that may avoid the need for more invasive cardiovascular or renal support.

## 5. Conclusions

Care must be taken when hyperinsulinaemia-euglycaemia is used to avoid hypoglycaemia. A trial of metaraminol early in severe cases of calcium channel blocker and angiotensin II receptor antagonist toxicity may be of benefit, especially when conventional ionotropic treatment measures are failing.

## Figures and Tables

**Table 1:** Initial effect of metaraminol boluses.

Time since admission to ED in hours	Metaraminol bolus of 0.5 mg	Systolic	Diastolic	MAP	Pulse
In ED		122	61	81	100
In ED 1 hour after admission		70	40	50	90
Taken from medical SHO clerking 3 hours after admission		75	43	54	96
5 when first seen by ICU		80	50	63	98
5 given metaraminol in boluses at intervals of 5–10 minutes.	0.5 mg + 0.5 mg	72	46	55	99
5:20		110	68	82	89
5 then given calcium gluconate (20 mLs of 10%) followed by glucagon 10 mg as a bolus	0.5 mg + 0.5 mg	125	62	84	100
5:30		120	74	89	86
6		90	50	63	85
7 noradrenaline started here		70	48	55	85
8		75	43	54	85
9		80	48	59	85
10		90	55	67	85
11—admitted to ICU- vasopressin started here along with glucagons infusion		85	47	60	95
12 adrenaline started here		95	50	65	80

**Table 2:** Initial blood gas results.

Time since admission to ED in hours	pH	BE	Bicarbonate	PCO_2_	PaO_2_	Lactate
In ED venous gas recorded	7.371	−2.3				
5 hours post ED	7.3	−3.9	21	7.49	7.9	
11:25—on trauma mask	7.34	−6.2	19.3	4.84	9.37	1.79
12:00—now on NIV	7.36	−5.8	19.5	4.52	7.83	2.5
13:00	7.3	−9.4	16.8	4.49	9.56	2.5
17:00	7.26	−10.4	16	4.7	9.43	6.44
18:00	7.27	−10.3	16.1	4.54	8.26	5.87
19:30	7.26	−11.2	15.5	4.5	10.00	6.25
20:54—bicarbonate started on basis of this gas—50 mLs/hr of 8.4% Total 200 mLs given	7.25	−13.3	14	3.95	10.89	7.78
22:54	7.31	−10.1	16.2	3.93	7.83	6.27
23:57	7.32	−8.0	17.9	4.53	9.47	4.23
25:00	7.35	−6.2	19.3	4.51	8.58	3.63
26:06	7.34	−6.0	19.5	4.96	10.08	2.89
27:01	7.33	−5.8	19.7	4.91	9.73	2.40
28:26	7.33	−5.7	19.7	4.97	9.64	2.05
29:32	7.34	−5.1	20.1	4.95	9.11	1.77
